# The Double-Edged Sword of AI Efficiency: Self-Efficacy Erosion as a Mediator Linking Instant Gratification and Perceived AI Efficacy to AI Dependency

**DOI:** 10.3390/bs16040530

**Published:** 2026-04-01

**Authors:** Xuehan Zhu, Aiai Zhang, Jiacheng Zhang

**Affiliations:** 1 School of Journalism and Communication, Tsinghua University, Beijing 100084, China; xuehanzhu@tsinghua.edu.cn (X.Z.); zaa24@mails.tsinghua.edu.cn (A.Z.); 2 School of Information, Central University of Finance and Economics, NO. 39, South Xueyuan Road, Haidian District, Beijing 100081, China

**Keywords:** AI dependency, instant gratification, perceived AI efficacy, self-efficacy erosion

## Abstract

Generative AI is becoming integral to daily workflows, fostering a novel form of functional cognitive AI dependency distinct from pathological addiction. While emerging research acknowledges this phenomenon, the specific psychological mechanisms underpinning its development remain underexplored. Incorporating self-efficacy erosion into the reinforcement-based framework, this study investigates whether instant gratification and perceived AI efficacy as key drivers of AI dependency. We examine the model using Structural Equation Modeling (SEM) with cross-sectional data collected from 576 users who have engaged with AI. The results show that both instant gratification and efficient rewards are positively associated with individuals’ AI dependency. Furthermore, users’ self-efficacy erosion significantly mediates the positive relation, supporting the hypothesis that greater reliance on AI is related to lower self-belief and stronger AI dependency. Moderation analyses further indicate that task-domain self-efficacy and social norms strengthen these positive associations. These findings provide empirical support for a mechanism associated with functional AI dependency and offer insights for navigating human–AI interaction while promoting balanced AI adoption.

## 1. Introduction

Generative Artificial Intelligence (AI) has rapidly evolved from a frontier innovation into a widely adopted cognitive tool embedded in daily work and learning practices. According to a report by McKinsey & Company (2023), one-third of organizations worldwide already routinely use generative AI in at least one business function, demonstrating an unprecedented pace and breadth of adoption. This profound transformation has given rise to a new paradigm of human–computer interaction, characterized by dynamics extending beyond mere tool usage and manifesting as a deep functional and cognitive dependency. Accumulating empirical evidence shows that excessive dependency on AI may undermine users’ internal cognitive capacities. For instance, studies across educational and professional domains reveal that heightened AI dependency is associated with a decline in self-confidence, independent problem-solving, critical thinking, analytical reasoning, and even professional competencies such as scientific writing and pedagogical engagement (Wijaya et al., 2024; Zhai et al., 2024; Alhur et al., 2025). These findings highlight a paradox, suggesting that technologies designed to enhance human capability may, through excessive or uncritical use, erode the very skills they aim to support. Against this backdrop, understanding the psychological mechanisms that drive AI dependency is becoming increasingly essential, particularly for guiding students and other frequent users toward forms of engagement that preserve autonomy and cognitive integrity.

Despite growing concerns surrounding AI dependency, the psychological foundations of this phenomenon remain insufficiently understood. Previous research on technology dependence often adopted a clinical psychology perspective, viewing it as a “pathological addiction” focused on users’ loss of control and has made valuable contributions by identifying the “push factors” of AI dependency (Lee, 1966), such as individual psychological vulnerabilities (e.g., neuroticism, low self-control) and situational pressures (e.g., academic stress) (Rodríguez-Ruiz et al., 2025; Zhang et al., 2024). While these studies help explain who may be pushed towards AI, they pay less attention to the “pull factors” inherent in the technology itself that make it compelling. Specifically, the mechanisms through which AI’s instantaneous and effective outputs are associated with psychological experiences such as instant gratification and perceived AI efficacy, which may in turn relate to AI dependency, remain empirically underexamined. Moreover, there is a lack of systematic research exploring how these pull factors interact with users’ internal cognitive beliefs, such as the erosion of self-efficacy, or how contextual forces such as task-domain efficiency and social norms of AI use may amplify or buffer these effects. This gap limits our understanding of the broader psychological landscape associated with AI dependency develops and highlights the need for a more integrative, mechanism-oriented investigation. This broader concern also resonates with emerging human-centred and human–AI interaction research, which suggests that users’ engagement with AI is shaped not only by functional utility but also by relational, perceptual, and social-cognitive factors (Shin et al., 2025).

To clarify the psychological mechanisms through which instant gratification and perceived AI efficacy foster AI dependency, we conducted a survey of 576 undergraduate and postgraduate students, which is an emerging demographic that increasingly engages with generative AI tools in both academic and daily cognitive tasks. Grounded in reinforcement-based perspectives and Social Cognitive Theory (Bandura, 1986, 1991), this study proposes a mechanism in which individuals’ preference for instant gratification and their perceived AI efficacy jointly shape their dependency on AI, with erosion of self-efficacy serving as a central mediating pathway. In addition, we examine whether this mechanism is contingent upon two boundary conditions: task-domain self-efficacy, representing users’ confidence in performing domain-specific tasks independently, and social norms of AI use, reflecting the perceived acceptability and prevalence of AI-assisted cognition within one’s social or academic environment.

By empirically testing this integrated framework, the study makes several contributions. First, it extends the existing research by moving beyond traditional “push factors” explanations to systematically examine the technological and experiential “pull factors” that may actively draw users toward AI dependency. Second, this study advances the theoretical understanding of AI efficacy by integrating the AI anthropomorphism framework. We conceptualize perceived AI efficacy as the attribution of competence to the system, which is a core dimension of anthropomorphic intelligence. This perspective clarifies the psychological nature of why users perceive AI as a superior cognitive agent rather than a passive tool. Third, by identifying erosion of self-efficacy as a mediating mechanism, the study illuminates an internal cognitive process through which efficiency-based reinforcement may be associated with weaker autonomous problem-solving tendencies. Third, by assessing the moderating roles of task-domain self-efficacy and social norms, the research delineates boundary conditions that clarify when and for whom these effects are most pronounced. Collectively, these contributions advance theoretical understanding of AI cognitive dependency and provide actionable insights for educators and policymakers aiming to promote healthy, autonomy-preserving patterns of AI use among students.

## 2. Theoretical Background and Hypotheses

### 2.1. AI Dependency

Distinct from traditional internet addiction, which emphasizes loss of control (e.g., Brand, 2022), AI dependency represents a functional pattern of reliance in which users increasingly incorporate generative AI into their routine cognitive activities and problem-solving processes. While AI offers substantial functional benefits, habitual offloading of cognitive tasks may gradually erode users’ internal competencies, underscoring the importance of understanding why dependency develops.

The existing literature has primarily focused on the determinants of AI dependency, with particular emphasis on “push factors” rooted in individual vulnerabilities. Personality traits such as neuroticism, impulsivity, and self-critical perfectionism have been found to elevate psychological need frustration and negative academic emotions, increasing tendencies to seek AI assistance as a compensatory coping strategy (Zhong et al., 2024). Similarly, deficits in self-control, self-esteem, or self-efficacy predict more frequent AI use, especially when students employ AI as a substitute for undertaking cognitively demanding tasks themselves (Rodríguez-Ruiz et al., 2025). Furthermore, situational pressures like academic stress play a critical catalytic role. The prior literature indicates that students with low academic self-efficacy are more susceptible to academic stress and hold higher performance expectancies towards AI; this stress and expectancy jointly mediate the pathway from low self-efficacy to high AI dependency (Zhang et al., 2024). While these studies offer valuable insights into who is more prone to AI dependency, they provide comparatively limited explanations of what features of AI itself draw individuals into habitual reliance. The reinforcing properties inherent in generative AI—such as immediacy, efficiency, and high perceived performance—remain less systematically examined, leaving open questions about how technological “pull factors” shape the development of AI cognitive dependency.

From a reinforcement-based perspective, behaviors are more likely to be repeated when they consistently produce valued outcomes, particularly when those outcomes are obtained quickly and with minimal cognitive effort. Generative AI exhibits these characteristics by delivering immediate responses, streamlining problem solving, and enabling users to achieve desired results with reduced time and mental effort. In this regard, prior work on reinforcer pathology offers a relevant theoretical lens, as it highlights how sensitivity to immediate and efficient rewards can shape recurring behavioral choices (Bickel et al., 2011). Applied to the present context, this perspective suggests that the attractiveness of generative AI may lie not only in users’ individual vulnerabilities, but also in the technology’s capacity to provide fast, low-effort, and reliably rewarding cognitive assistance.

### 2.2. Instant Gratification and AI Dependency

Instant gratification refers to individuals’ preference for immediate, low-effort rewards over delayed, effortful outcomes (Torrubia et al., 2001; McClure et al., 2004). This tendency is closely related to temporal reward discounting and can be understood in light of reinforcer pathology, which highlights the strong appeal of immediately available rewards in shaping repeated behavioral choices (Bickel et al., 2011). Although this framework was originally developed in the context of maladaptive behaviors, its core insight regarding sensitivity to immediacy remains relevant for understanding technology use in digital environments characterized by rapid and streamlined forms of reward (Brook & Santos, 2023).

Generative AI aligns closely with users’ preference for instant gratification. It delivers immediate, relevant responses that bypass the temporal and cognitive delays inherent in traditional problem-solving. Instead of engaging in prolonged reasoning, information search, or iterative revision, users can receive high-quality outputs almost instantly. This immediacy reduces cognitive friction and produces a rewarding sense of efficiency, making AI a powerful source of rapid reinforcement. On the one hand, repeated satisfactory outcomes obtained through AI may reinforce the tendency to rely on it in the future. On the other hand, the ease and immediacy of AI-assisted completion may weaken users’ willingness to sustain the self-control and effort required for independent problem-solving, making it harder to resist turning to AI when facing cognitive demands (Duckworth et al., 2016). These reinforcing and self-regulatory processes may jointly increase the likelihood of AI dependency.

Thus, individuals who strongly prefer immediate rewards may be more inclined to rely on AI, because AI-generated solutions are especially attractive to those who value speed and low-effort task completion. This tendency may be associated with greater reliance on AI and, consequently, with higher levels of AI dependency. Therefore, we hypothesize:

**H1.**  *Preference for instant gratification is positively associated with AI dependency.*

### 2.3. Perceived AI Efficacy and AI Dependency

While instant gratification represents the temporal mechanism of dependence, perceived AI efficacy reflects the substantive mechanism behind users’ reliance on AI. Prior research has identified technological readiness as a precursor of AI use (Anh & Duc, 2024). As users gain experience with generative AI, they may increasingly perceive it as an effective tool for producing efficient outputs and enhancing productivity (Brynjolfsson et al., 2025). Building on this line of research, we focus on perceived AI efficacy, namely users’ belief that AI can effectively improve task performance and problem-solving efficiency. From a reinforcement-based perspective, such perceptions function as a form of efficiency reward, i.e., users repeatedly experience that relying on AI yields successful outcomes with substantially reduced cognitive effort. In this study, we conceptualize this experience of efficient rewards as the perceived AI efficacy.

Meanwhile, perceived AI efficacy may reflect more than a purely instrumental evaluation of usefulness. It can also be understood through the lens of AI anthropomorphism. According to the integrative framework proposed by Curșeu and Radu (2026), competence is a central dimension of anthropomorphic perception. In this sense, users’ belief that AI can effectively solve problems and improve performance reflects the extent to which they attribute competence to AI as an intelligent agent. When AI is perceived not merely as a passive tool but as a highly competent partner, users may become more willing to delegate cognitive tasks to it and incorporate it into their routine problem-solving processes.

This competence-based perception helps explain why perceived AI efficacy may foster stronger AI dependency. When users perceive AI to be highly effective, each positive interaction strengthens the expectancy that AI use enhances task performance, thereby increasing the motivation to rely on AI in subsequent tasks. Repeated efficiency-based reinforcement may also encourage users to incorporate AI more deeply into their cognitive routines, making externally assisted processing more attractive than independent problem-solving. As beliefs in AI’s capability become stronger, AI may come to be seen not merely as a tool but as a reliable shortcut for achieving academic or cognitive goals. Consistent with research on cognitive offloading, tools perceived as efficient are more readily integrated into habitual cognitive routines (Sparrow et al., 2011; Risko & Gilbert, 2016). Therefore, the more users believe in the AI’s efficacy, the more likely they are to rely on it to complete tasks, thus forming a self-reinforcing cycle of dependency. Accordingly, we propose Hypothesis 2:

**H2.**  *Perceived AI efficacy is positively associated with AI dependency.*

### 2.4. The Mediation Role of Erosion of Self-Efficacy

Self-efficacy, defined as individuals’ beliefs in their capability to perform tasks successfully (Bandura, 1986), plays a fundamental role in shaping motivation, effort investment, and persistence. Social Cognitive Theory (SCT) emphasizes that self-efficacy is dynamically shaped through mastery experiences, social persuasion, and performance feedback. When individuals rely on external aids to complete cognitively demanding tasks, they may experience fewer opportunities for mastery and reduced internal attribution of competence. In the context of generative AI, repeated exposure to rapid, high-quality outputs can shift users’ perceptions of task success from internal ability to external assistance. This pattern gradually undermines users’ confidence in their own cognitive capabilities, thereby contributing to an erosion of self-efficacy (Zhong et al., 2024; Rodríguez-Ruiz et al., 2025).

This erosion, in turn, may increase users’ reliance on AI. SCT posits that individuals with lower self-efficacy are more likely to avoid challenging tasks, depend on external support, and exhibit reduced autonomous problem-solving behaviors. When users begin to doubt their own ability to complete tasks independently, AI becomes an appealing compensatory mechanism that minimizes perceived risk and effort. Thus, self-efficacy erosion serves as a psychological pathway linking reinforcement-driven tendencies, such as the desire for instant rewards and perceptions of AI’s superior performance, to the development of AI cognitive dependency. Across repeated cycles of outsourcing and diminished confidence, individuals may become increasingly reliant on AI as a primary means of handling academic or cognitive tasks. Therefore, we propose:

**H3.**  *Erosion of self-efficacy mediates the positive relationship between preference for instant gratification and AI dependency.*

**H4.**  *Erosion of self-efficacy mediates the positive relationship between perceived AI efficacy and AI dependency.*

### 2.5. The Moderating Effect of Task-Domain Self-Efficacy

Task-domain self-efficacy refers to an individual’s belief in their capability to perform successfully in a specific domain where generative AI is used (Venkatesh et al., 2003). In traditional social-cognitive accounts, higher self-efficacy is often associated with greater autonomy and persistence. However, in AI-assisted environments, high task-domain self-efficacy may also increase a user’s ability and willingness to incorporate external tools into task execution when such tools are perceived as useful, efficient, and performance-enhancing. Rather than relying on AI because they feel incapable, highly self-efficacious users may rely on it because they are better able to recognize where AI adds value, to direct it strategically, and to integrate it effectively into their workflow. As a result, AI use may become more frequent, instrumental, and routinized among these users.

This logic suggests that task-domain self-efficacy can strengthen the effects of both instant gratification preference and perceived AI efficacy on AI dependency. For individuals with stronger instant gratification tendencies, high self-efficacy may facilitate the effective use of AI to obtain fast and satisfactory outcomes, thereby reinforcing repeated reliance. Likewise, when users with high task-domain self-efficacy also perceive AI as accurate, trustworthy, and efficient, they may be especially likely to embed it into their regular task practices as a dependable cognitive partner. In this sense, self-efficacy does not necessarily reduce AI dependency; instead, it may amplify dependency by enabling more confident, skilled, and sustained integration of AI into domain-specific work. Therefore, we propose:

**H5a.**  *Task-domain self-efficacy positively moderates the relationship between preference for instant gratification and AI dependency.*

**H5b.**  *Task-domain self-efficacy positively moderates the relationship between perceived AI efficacy and AI dependency.*

### 2.6. The Moderating Effect of Social Norms

Social norms reflect the perceived social pressure and expectations regarding the use of technology within a relevant group (Venkatesh et al., 2003). In environments where using generative AI is common, encouraged, or seen as a marker of efficiency, individual perceptions and preferences are likely to translate into dependent behaviors. We propose that strong social norms favoring AI use will amplify the positive effects of the predictors (X_InstGrat and X_Efficacy) on AI dependency. When the social context normalizes and validates reliance on AI, individuals with a high preference for Instant Gratification, a supportive social norm reduces potential barriers or self-regulatory efforts needed to resist the immediate rewards offered by AI. Likewise, individuals who perceive AI as highly effective may feel less internal or external friction in deepening their usage, strengthening the positive association with AI dependency. The environment lowers the threshold for acting on the preference for immediacy through AI use, thereby strengthening the link between this preference and dependency. Thus, we have Hypothesis 6:

**H6a.**  *Social norms of AI use positively moderate the relationship between preference for instant gratification and AI dependency.*

**H6b.**  *Social norms of AI use positively moderate the relationship between perceived AI efficacy and AI dependency.*

**Figure 1:**
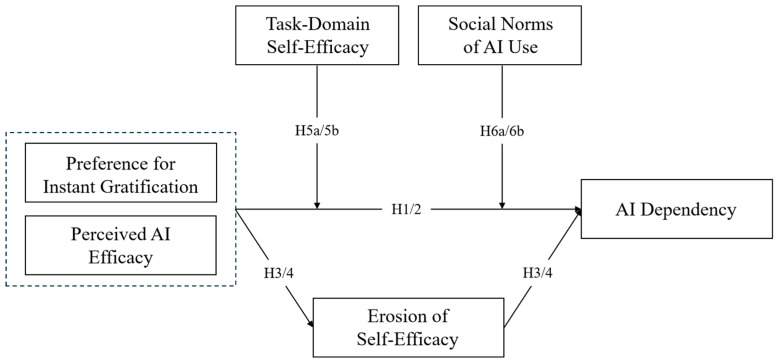
Proposed research model.

In summary, the research model of this study is shown in Figure 1.

## 3. Data, Method, and Validation

### 3.1. Questionnaire Participants, Measurement of Variables, and Reliability Analysis

In this study, we employed a questionnaire survey targeting undergraduate and postgraduate students. This group was selected for two primary reasons. First, they are among the earliest adopters and most frequent users of generative AI in academic and cognitive tasks, making them ideal for exploring emerging patterns of AI cognitive dependency. University students increasingly integrate generative AI into learning, writing, and problem-solving activities (Kasneci et al., 2023), often relying on AI for rapid feedback and efficient task completion. This population thus provides a critical context for examining how instant gratification and perceived AI efficacy interact to shape functional and cognitive reliance on AI tools. Second, they represent key stages in academic development where research skills, attitudes, and critical thinking are actively formed. Undergraduate students are building foundational academic and research abilities, while postgraduates are refining these through independent inquiry and thesis work. Studying both groups enables comparison across levels of research experience and provides insights applicable to higher education improvement (Adebisi, 2022; Daniel, 2022).

All items in this study were measured using a 5-point Likert scale ranging from 1 (totally disagree) to 5 (totally agree), unless otherwise specified. The measurement instruments were primarily adapted from previously validated scales found in well-documented literature to ensure a foundation of reliability and validity. A total of 751 questionnaires were distributed and collected. After excluding responses from participants who did not meet the study’s target criteria (e.g., non-students or incomplete submissions), 576 valid responses were collected, comprising 469 undergraduate and 107 postgraduate students. Among the respondents, 30% were male and 70% were female, with ages ranging from 18 to 25 years. Detailed descriptions of the measurement variables and the results of reliability analyses are presented below and Table 1 Column (1).

#### 3.1.1. AI Dependency Scale (ADS-R)

This scale measures the core dependent variable, AI_Dependency (Y), defined as a functional and cognitive reliance on generative AI tools rather than pathological addiction. The initial item pool was adapted from the classic 20-item Internet Addiction Test (IAT) by Young (1998), considering the psychometric analysis by Widyanto and McMurran (2004) and contextualizing items for generative AI use. Exploratory and Confirmatory Factor Analyses (EFA/CFA) on the current sample revealed a clear and robust two-factor structure underlying AI dependency, deviating from the originally adapted multi-dimensional framework. The final scale used for analysis consists of 15 items loading onto two distinct factors. Specifically, Y_FunctionalDep (Factor 1): Cognitive Integration and Habit (9 items, e.g., “I feel AI has become an indispensable part of my knowledge system or external brain”) and Y_AutonomyLoss (Factor 2): Autonomy Erosion and Life Proxy (6 items, e.g., “People around me have commented that I can’t work/think without AI”). Scores for each subscale (and an overall dependency score) were calculated by averaging the responses to the corresponding items. A higher score indicates greater AI dependency. Cronbach’s α in this study was excellent for Factor 1 (α = 0.91), Factor 2 (α = 0.89), and the overall 15-item scale (α = 0.92).

#### 3.1.2. Preference for Instant Gratification (X_InstGrat)

This scale measures the stable individual trait of preferring immediate rewards over larger, delayed ones. The 4 items were developed based on the core concepts related to reward sensitivity in the Sensitivity to Punishment and Sensitivity to Reward Questionnaire (SPSRQ) by Torrubia et al. (2001) (e.g., “Compared to things that require patience for a larger reward, I prefer things that yield immediate results”). The score was calculated by averaging the responses. A higher score indicates a stronger preference for Instant Gratification. Cronbach’s α in this study was acceptable at 0.72.

#### 3.1.3. Perceived AI Efficacy (X_Efficacy)

This scale measures the user’s subjective evaluation of generative AI’s ability to efficiently provide high-quality solutions. This construction was measured using an 8-item scale adapted from the UTAUT (Venkatesh et al., 2003). Example items include, “I believe AI tools provide high-quality solutions and information” and “Overall, I find AI tools very useful in my work/study”. The score was calculated by averaging the responses, with a higher score indicating greater perceived AI efficacy. Cronbach’s α in this measure was excellent at 0.90.

#### 3.1.4. Erosion of Self-Efficacy (M_Erosion)

This scale measures the extent to which users perceive a decline in their confidence to perform tasks independently as a result of relying on AI. The 4 items were developed based on the concept of self-efficacy from Social Cognitive Theory (Bandura, 1986), with item phrasing adapted in a reverse and contextualized manner, drawing inspiration from measurement approaches related to self-efficacy in technology use contexts (e.g., Venkatesh et al., 2003) (e.g., “As I get more used to using AI, I feel my ability to think independently and solve problems has somewhat declined”). The score was calculated by averaging the responses. A higher score indicates a greater perceived erosion of self-efficacy. Cronbach’s α in this study was good at 0.83.

#### 3.1.5. Task-Domain Self-Efficacy (Mo_TaskSE)

This scale measures the user’s baseline confidence in their ability to perform tasks within the specific domain where they most frequently utilize generative AI. The 3 items were adapted from the self-efficacy scale used by Venkatesh et al. (2003) and participants were instructed to answer based on their primary AI application area (e.g., “In this domain, I am very confident in my ability to complete tasks independently”). The score was calculated by averaging the responses. A higher score indicates higher self-efficacy in the relevant task domain. Cronbach’s α in this study was good at 0.82.

#### 3.1.6. Social Norms of AI Use (Mo_SocialNorms)

This scale measures the perceived social influence and prevailing attitudes towards using generative AI within the user’s relevant social environment (e.g., team, organization). The 3 items were adapted from the “Social Influence” dimension of the UTAUT model (Venkatesh et al., 2003) (e.g., “My classmates extensively use AI tools to assist their work/study”). The score was calculated by averaging the responses. A higher score indicates stronger perceived social norms favoring AI adoption and use. Cronbach’s α in this study was acceptable at 0.77.

### 3.2. Confirmatory Factor Analysis of the Measurement Model

To further assess the adequacy of the measurement structure, we conducted confirmatory factor analysis (CFA) for the focal constructs. The full measurement model, including the two AI dependency dimensions, perceived AI efficacy, erosion of self-efficacy, instant gratification, task-domain self-efficacy, and social norms, demonstrated acceptable overall fit, with RMSEA = 0.06, CFI = 0.89, TLI = 0.88, and SRMR = 0.06. All standardized factor loadings were statistically significant and generally moderate to high, supporting the adequacy of the proposed latent-factor structure.

To further reduce concerns about potential overfitting from relying on a single sample, we conducted an additional split-sample validation procedure. The full sample was randomly divided into two equal halves. Using the second subsample as a validation sample (N = 288), we re-estimated the CFA measurement model for the focal constructs. The results were broadly consistent with those obtained from the full sample, with acceptable overall fit (RMSEA = 0.07, CFI = 0.88, TLI = 0.87, SRMR = 0.07), and all standardized factor loadings remained statistically significant and substantively similar. Taken together, these findings suggest that the proposed measurement structure is reasonably stable and that the focal constructs can be modeled as related but conceptually distinguishable latent variables in the subsequent structural analyses.

## 4. Results

### 4.1. Summary Statistics

Table 1 Column (2) presents the means, standard deviations, minimum, and maximum values for the key constructs utilized in this study. All constructs were measured using items rated on a 5-point Likert scale, ranging from 1 (totally disagree) to 5 (totally agree).

The mean score for overall AI dependence (AI_Dependency) is 2.92, a value slightly below the scale’s neutral midpoint of 3 (on a 1–5 scale). This suggests that, on average, participants leaned slightly towards disagreeing with statements characterizing AI dependence. Examining the two sub-dimensions revealed nuances. Functional Dependency (Y_FunctionalDep) has a mean of 3.223, which is slightly above the neutral point. This indicates that, on average, participants tended to agree more with statements reflecting AI’s integration into their cognitive processes and work habits. In contrast, Autonomy Loss (Y_AutonomyLoss) has a mean of 2.635, slightly below the neutral point, suggesting less agreement with statements reflecting a loss of independence or delegation of life tasks to AI.

Furthermore, the means for both Perceived AI Efficacy (X_Efficacy, M = 3.492) and Preference for Instant Gratification (X_InstGrat, M = 3.509) are above the neutral point. This indicates that most participants generally perceive AI tools as effective and trustworthy and also tend to possess a preference for immediate outcomes.

### 4.2. Pearson Correlation

Next, we examine the correlation among the variables. The results in Table 1 Column (3) show that the overall AI dependence (AI_Dependency) is positively and significantly related to our key independent variables (X_InstGrat and X_Efficacy), providing preliminary evidence of positive associations between these variables and participants’ AI dependence.

### 4.3. Path Analysis Baseline Results

Before testing the hypotheses, the overall model fit is evaluated. The structural model for instant gratification (*X_InstGrat*) shows acceptable fit to the data (CFI = 0.922, TLI = 0.912, RMSEA = 0.067, SRMR = 0.067). Similarly, the model for perceived AI efficacy (*X_Efficacy*) demonstrates acceptable fit statistics (CFI = 0.902, TLI = 0.891, RMSEA = 0.072, SRMR = 0.069). These indices confirm that the proposed structural models provide a robust framework for testing the hypothesized relationships.

#### 4.3.1. Key Independent Variable 1: Instant Gratification (X_InstGrat)

The results of the structural equation modeling (SEM) analysis for the effects of X_InstGrat (i.e., Instant Gratification Preference) are presented in Table 2. Specifically, Table 2 Columns (1)–(2) show that X_InstGrat has a significant positive effect on Y_FunctionalDep (β = 0.520, z = 13.57) and Y_AutonomyLoss (β = 0.515, z = 10.08), confirming that technological “pull factors”, i.e., X_InstGrat function, play as potent cognitive shortcuts. These results are consistent with the argument that, from the perspective of reinforcer pathology, the rapid and low-effort rewards associated with AI use may be linked to stronger habitual reliance, reduced engagement in traditional problem-solving, and higher reported autonomy loss.

Furthermore, Column (3) indicates that X_InstGrat significantly predicts M_Erosion (β = 0.589, z = 12.95), suggesting that Instant Gratification preference is positively associated with the erosion of self-efficacy. From a Social Cognitive Theory perspective, this pattern may reflect reduced opportunities for effortful mastery experiences, which are typically important for maintaining confidence in an individual’s own capabilities.

In the final path to AI_Dependency, Column (4) shows that X_InstGrat has a direct positive effect on AI_Dependency (β = 0.234, z = 6.28), and M_Erosion exerts a positive influence on AI_Dependency (β = 0.481, z = 15.97). To formally test the mediation hypothesis, we employed the bootstrapping method with 5000 resamples. The results show a significant indirect effect of X_InstGrat on AI_Dependency through M_Erosion (indirect effect = 0.28, SE = 0.04, z = 7.89). The 95% bias-corrected confidence interval [0.213, 0.353] does not include zero, supporting a partial mediation model. This mediation pattern is consistent with the possibility that stronger instant gratification preference is associated with greater AI dependency partly through higher reported erosion of self-efficacy. One possible interpretation is that users who prioritize rapid AI outputs may engage less frequently in effortful cognitive challenges that support a sense of internal mastery, and this pattern may in turn be linked to greater reliance on AI.

Overall, the findings indicate that instant gratification preference is associated with AI dependency both directly and indirectly via the erosion of self-efficacy, with the indirect pathway appearing more pronounced.

#### 4.3.2. Key Independent Variable 2: Perceived AI Efficacy (X_Efficacy)

The results of the SEM analysis for the effects of X_Efficacy (Perceived efficacy and trust) are presented in Table 2. Specifically, Table 2 Columns (5)–(6) show that X_Efficacy has a strong and significant positive effect on Y_FunctionalDep (β = 0.697, z = 19.45) and Y_AutonomyLoss (β = 0.558, z = 10.50), indicating that individuals with higher perceived efficacy in and trust toward AI tend to report greater functional dependence on AI and higher levels of autonomy loss. Theoretically, these results suggest that users’ trust in AI’s superior performance may function as an “efficiency reward.” Repeated successful outcomes with relatively low effort may be associated with stronger perceptions of AI indispensability and greater reliance on AI in decision-related tasks.

Furthermore, Column (7) demonstrates that X_Efficacy significantly predicts M_Erosion (β = 0.612, z = 12.84), suggesting that perceived efficacy and trust is positively associated with the erosion of self-efficacy. This pattern may indicate that when AI is perceived as highly effective, users may be more likely to attribute successful task performance to the tool rather than to their own skills, which may be associated with greater erosion of self-efficacy.

In the final path to AI_Dependency, Column (8) shows that X_Efficacy has a direct positive effect on AI_Dependency (β = 0.357, z = 9.55), and M_Erosion exerts a positive influence on AI_Dependency (β = 0.441, z = 15.27). To validate the mediating mechanism, we performed a bootstrapping analysis with 5000 resamples. The results confirm a significant indirect effect of X_Efficacy on AI_Dependency via M_Erosion (Indirect Effect = 0.270, SE = 0.037, z = 7.36). The 95% bias-corrected confidence interval [0.198, 0.341] does not include zero, supporting a partial mediation model. This mediation pattern is consistent with the possibility that perceived AI efficacy is associated with greater AI dependency partly because stronger confidence in the tool (i.e., higher competence attribution) may coincide with lower confidence in one’s own independent capability.

Overall, the findings indicate that perceived efficacy and trust are associated with AI dependency both directly and indirectly via the erosion of self-efficacy, with the indirect pathway appearing more pronounced.

### 4.4. Moderating Test Results

While the direct and mediated pathways outline the core mechanism, the strength of these relationships may not be uniform across all individuals and contexts. We propose that individual differences in task-specific confidence and variations in the social environment act as crucial boundary conditions, moderating the extent to which instant gratification preference and perceived efficacy are associated with AI dependency.

#### 4.4.1. Moderator 1: Task-Specific Self-Efficacy (Mo_TaskSE)

**Figure 2:**
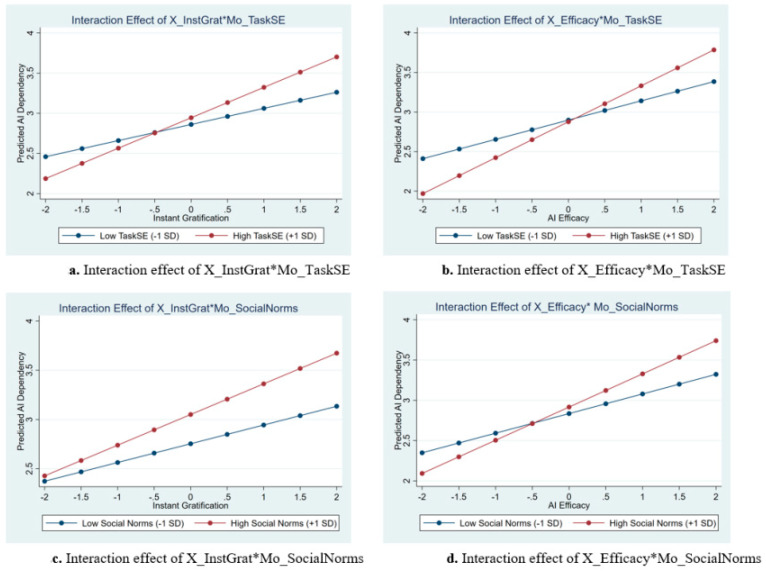
Interaction effects of predictors and moderators on AI dependency. Panels (**a**,**b**) illustrate the moderating role of Task-Specific Self-Efficacy; panels (**c**,**d**) illustrate the moderating role of Social Norms. * is the interaction sign.

We first examine whether task-specific self-efficacy (Mo_TaskSE) can moderate the relation between the predictors (X_InstGrat and X_Efficacy) and AI dependency. Table 3 Columns (1)–(2) present the moderation analysis results for the moderating role of Mo_TaskSE in the relationships between core predictors (X_InstGrat and X_Efficacy) and AI_Dependency. In particular, Column (1) shows that the interaction term X_InstGrat*Mo_TaskSE shows a significant positive effect on AI_Dependency (β = 0.089, z = 4.95), indicating that higher task-specific self-efficacy strengthens the positive impact of Instant Gratification preference on AI dependency. As visualized in the simple slope plot (Figure 2a), the positive relationship between instant gratification and AI dependency is steeper for users with high task-specific self-efficacy than for those with low self-efficacy. One possible interpretation is that more confident users may be better able to use AI efficiently to obtain rapid outcomes, thereby making instant gratification more closely linked to dependency tendencies.

Moreover, Column (2) shows that the interaction term X_Efficacy*Mo_TaskSE also exhibits a significant positive effect on AI_Dependency (β = 0.105, z = 6.31), suggesting higher task-specific self-efficacy amplifies the positive association between perceived AI efficacy and AI dependency. Figure 2b similarly shows that the positive association between perceived AI efficacy and AI dependency is stronger among users with higher task-specific self-efficacy. This pattern may reflect that confident users are more likely to integrate AI into their task processes when they also perceive the tool as effective and trustworthy.

Overall, these results suggest that AI dependency may be associated with strategic usage patterns among capable users, rather than reflecting only compensatory reliance among less skilled individuals. The results are consistent with our predictions.

#### 4.4.2. Moderator 2: Social Norms of AI Use (Mo_SocialNorms)

Next, we employ social norms (Mo_SocialNorms) as a moderator to test Hypothesis 6. Table 3 Columns (3)–(4) present the moderation analysis results for the moderating role of Mo_SocialNorms in the relationships between predictors (X_InstGrat and X_Efficacy) and AI_Dependency. Specifically, Column (3) shows that the interaction term X_InstGrat*Mo_SocialNorms shows a significant positive effect on AI_Dependency (β = 0.061, z = 3.41), indicating that stronger social norms strengthen the positive association between instant gratification preference and AI dependency. The visualization in Figure 2c demonstrates that a supportive social environment strengthens the link between gratification preference and dependency. These results suggest that a supportive social environment may lower the self-regulatory barriers to seeking instant AI rewards. When AI use is more normalized, prioritizing speed over independent effort may become easier and more socially acceptable.

Moreover, Column (4) demonstrates that the interaction term X_Efficacy* Mo_SocialNorms also exhibits a significant positive effect on AI_Dependency (β = 0.084, z = 4.94), suggesting that stronger social norms amplify the positive association between perceived efficacy and AI dependency. As shown in Figure 2d, the slope representing the effect of perceived efficacy on dependency is noticeably steeper in high-norm environments. These results suggest that trust in AI’s efficiency may be socially validated in high-norm environments, making individual reliance on AI more consistent with prevailing group expectations.

In summary, social norms appear to function as an environmental amplifier, strengthening the positive associations between the focal predictors and AI dependency. Overall, the results are consistent with our predictions.

### 4.5. Robustness Check

To avoid the baseline results are driven by omitted variables, we additionally add *Male* as a control variable, an indicator variable equals 1 if the respondent is male and 0 otherwise. This is motivated by prior literature suggesting that gender differences may be associated with distinct patterns of technology-related dependency or usage behavior (Venkatesh et al., 2003). Table 4 presents the SEM results for these robustness checks. Column (1) examines the instant gratification (*X_InstGrat)* effect. The results indicate that *X_InstGrat* continues to exert a significant positive effect on both *M_Erosion* (β = 0.597, z = 13.08) and *AI_Dependency* (β = 0.225, z = 6.00). Gender (*Male*) shows a slight positive correlation with AI dependency in this path, suggesting that male users are more likely to rely on AI relative to females. This pattern is consistent with prior evidence showing that males tend to exhibit greater vulnerability to internet addiction-related tendencies than females (Su et al., 2019).

Column (2) presents the results for perceived AI efficacy (*X_Efficacy*). Similarly, the effects of *X_Efficacy* on *M_Erosion* (β = 0.621, z = 12.97) and *AI_Dependency* (β = 0.349, z = 9.27) remain robust. In this model, the effect of gender on the outcome variables is not statistically significant, suggesting that the primary drivers of AI dependency in our model are cognitive and psychological factors rather than demographic differences. Overall, the coefficients of the focal variables remain stable and significant across both models in Table 4, confirming that our baseline findings are robust to the inclusion of gender-related controls.

## 5. Discussion

Generative AI’s rapid integration into daily life has spurred a new form of functional cognitive dependency, distinct from traditional pathological addiction and driven significantly by efficiency gains. This study aims to fill a critical gap in understanding the antecedents of this AI dependency by investigating the roles of instant gratification preference and perceived AI efficacy. Surveying 576 university students and employing structural equation modeling, our research empirically tested a moderated mediation model based on these core drivers.

The findings confirm that both users’ intrinsic preference for instant gratification and their positive evaluation of AI’s efficacy and trustworthiness are significant positive predictors of overall AI dependency. This supports our central thesis that the combination of satisfying users’ desire for instant results and providing efficient, high-quality rewards acts as a key engine driving reliance on generative AI tools. Furthermore, the results reveal that the erosion of self-efficacy significantly mediates the relationship between both predictors and AI dependency, highlighting that AI’s attractive, i.e., perceived power and speed, can contribute to a decline in users’ confidence in their own abilities, thereby reinforcing dependence.

Moreover, the study identified boundary conditions. Both task-domain self-efficacy and social norms favoring AI use can amplify the positive relation, suggesting that individual confidence and the surrounding social environment play crucial roles in shaping how user preferences and perceptions translate into dependent behaviors. These findings remained robust after controlling for gender.

### 5.1. Theoretical Implications

This study makes several contributions to the nascent literature on AI cognitive dependency. First, this paper provides a more nuanced understanding that moves beyond traditional pathological addiction frameworks. Our findings highlight how the pursuit of efficiency and immediate cognitive closure can become potent drivers of reliance. Specifically, the identification of self-efficacy erosion as a key mediating pathway offers empirical support for conceptualizations of AI dependency as a form of cognitive offloading with potential downsides (Sparrow et al., 2011), linking positive AI perceptions (efficacy/trust) to potential cognitive risks such as diminished confidence in independent abilities (Carr, 2020; Ward et al., 2017).

Second, our findings provide empirical support for the AI anthropomorphism framework. As per Curșeu and Radu (2026), perceived AI efficacy represents the attribution of competence to the system, which is a core dimension of anthropomorphism. This results in a competence-driven dependency aligned with Proposition 2 of their framework, where the perceived superiority of the AI agent overshadows human agency and facilitates the observed erosion of self-efficacy.

Third, our results suggest that for generative AI, users’ valuations of performance benefits may be inextricably linked with their trust in the tool’s outputs. This highlights a potential need for refinement to traditional technology acceptance models (e.g., UTAUT, TAM) to better capture the unique interplay of these perceptions in the context of advanced AI tools.

### 5.2. Practical Implications

This study also has practical implications. Particularly, the findings offer insights for educators, students, and AI developers. For educators and institutions, recognizing that dependency can stem from efficiency-seeking highlights the need for strategies beyond simple restriction. Interventions should focus on promoting metacognitive awareness about reliance patterns and fostering critical evaluation skills to counteract potential deskilling or cognitive complacency. Educational guidelines could explicitly address the self-efficacy erosion mechanism, encouraging students to consciously balance AI assistance with independent practice. For students, understanding that both their intrinsic preference for immediacy and positive perceptions of AI can lead to dependence via self-efficacy erosion empowers them to adopt more mindful usage patterns, perhaps deliberately choosing tasks for independent completion. For AI developers, the findings suggest potential avenues for designing “healthier” AI interactions. Interfaces could incorporate features that manage user expectations for instant gratification (e.g., introducing slight delays for complex queries, explicitly stating confidence levels) or prompt users to reflect on AI outputs, potentially mitigating cognitive offloading risks and bolstering user self-efficacy.

### 5.3. Limitations and Future Research

While this study provides valuable insights into the mechanisms driving AI cognitive dependency, several limitations should be acknowledged, which also point towards avenues for future research. First, while our reliance on a student sample necessitates caution regarding generalizability, future work should examine diverse populations. Second, the cross-sectional design precludes definitive causal claims. Although the proposed mediation model is theoretically grounded, alternative directional relationships remain possible. For example, self-efficacy erosion may also shape individuals’ instant gratification preference or perceived AI efficacy. Nevertheless, the observed relationships remain consistent with the proposed framework and thus provide meaningful initial evidence for understanding how these factors are linked to AI dependency. Future longitudinal and experimental studies are needed to establish the causal ordering of these relationships. Third, an important limitation concerns the measurement of AI dependency. Although the adapted scale used in this study demonstrated satisfactory reliability and a clear factor structure in the current sample, it was derived from the Internet Addiction Test, which was originally developed to assess problematic internet overuse characterized by compulsivity and functional impairment. Accordingly, the present measure should be regarded as an adapted indicator of AI dependency rather than a fully established AI-specific instrument. Future research is encouraged to employ or compare more explicitly validated AI dependency measures (Goh et al., 2025). Finally, future studies could explore additional antecedents and boundary conditions associated with AI dependency, including interaction-related factors such as anthropomorphism (Curșeu & Radu, 2026), while also designing and testing interventions that promote more mindful AI use, thereby building a more comprehensive understanding of this emerging phenomenon.

## Figures and Tables

**Table 1:** Reliability Analysis, Summary Statistics, and Pearson Correlation Matrix.

	(1)	(2)		(3)					
Variables	Cronbach’s α	Mean	Std. Dev.	1	2	3	4	5	6
1 AI_Dependency	0.92	2.929	0.696	1					
2 X_Efficacy	0.9	3.492	0.617	0.555 *	1				
3 X_InstGrat	0.72	3.509	0.645	0.480 *	0.599 *	1			
4 M_Erosion	0.83	3.066	0.8	0.656 *	0.472 *	0.475 *	1		
5 Mo_TaskSE	0.82	3.432	0.647	0.235 *	0.396 *	0.126 *	0.300 *	1	
6 Mo_SocialNorms	0.77	3.632	0.601	0.389 *	0.632 *	0.384 *	0.443 *	0.442 *	1

Table 1 Column (1) displays Cronbach’s α coefficients indicating the internal consistency reliability for each scale used in the study. Column (2) presents all variables’ summary statistics. All variables represent mean scores calculated from items measured on a 5-point Likert scale (1 = Totally disagree, 5 = Totally agree). Column (3) presents all variables’ Pearson Correlation Matrix. AI_Dependency is overall AI dependency. *AI_Dependency* is overall AI dependency; *X_Efficacy* is perceived AI efficacy; *X_InstGrat* is preference for Instant Gratification; *M_Erosion* is the mediator, erosion of self-efficacy; *Mo_TaskSE* and *Mo_SocialNorms* are moderators, task-domain self-efficacy and social norms of AI use, respectively. Significance at the 10% level is indicated by *.

**Table 2:** Path Analysis Baseline Results.

	(1)	(2)	(3)	(4)	(5)	(6)	(7)	(8)
	Y_FunctionalDep	Y_AutonomyLoss	M_Erosion	AI_Dependency	Y_FunctionalDep	Y_AutonomyLoss	M_Erosion	AI_Dependency
X_InstGrat	0.520 ***	0.515 ***	0.589 ***	0.234 ***				
	(13.57)	(10.08)	(12.95)	(6.28)				
X_Efficacy					0.697 ***	0.558 ***	0.612 ***	0.357 ***
					(19.45)	(10.50)	(12.84)	(9.55)
M_Erosion				0.481 ***				0.441 ***
				(15.97)				(15.27)
N	576	576	576	576	576	576	576	576

Table 2 displays standardized path coefficients (β) from SEM predicting AI Dependency. Columns (1)–(2) present the influence of an individual’s preference of Instant Gratification (*X_InstGrat*) on *Y_FunctionalDep* (functional dependency/cognitive integration) and *Y_AutonomyLoss* (autonomy loss/life proxy). Column (3) provides the results of the effect of an individual’s preference of Instant Gratification on the mediator erosion of self-efficacy (*M_Erosion*). Column (4) shows the effects of *X_InstGrat* and *M_Erosion* on *AI_Dependency* from the mediation model. Columns (5)–(6) present the influence of an individual’s perceived AI efficacy (*X_Efficacy*) on *Y_FunctionalDep* (functional dependency/cognitive integration) and *Y_AutonomyLoss* (autonomy loss/life proxy). Column (7) provides the results of the effect of an individual’s perceived AI efficacy on the mediator erosion of self-efficacy (*M_Erosion*). Column (8) shows the effects of *X_Efficacy* and *M_Erosion* on *AI_Dependency* from the mediation model. *AI_Dependency* is overall AI dependency; Y_FunctionalDep is functional dependency/cognitive integration; *Y_AutonomyLoss* is autonomy loss/life proxy; *X_Efficacy* is perceived AI efficacy; *X_InstGrat* is preference for Instant Gratification; *M_Erosion* is the mediator, erosion of self-efficacy. Significance at the 1% level is indicated by ***.

**Table 3:** Moderating tests.

	(1)	(2)	(3)	(4)
	AI_Dependency	AI_Dependency	AI_Dependency	AI_Dependency
X_InstGrat	0.29 *** (10.96)		0.251 *** (9.04)	
X_Efficacy		0.349 *** (13.45)		0.328 *** (10.54)
Mo_TaskSE	0.042 (1.57)	−0.01 (−0.40)		
Mo_SocialNorms			0.148 *** (5.41)	0.04 (1.33)
X_InstGrat*Mo_TaskSE	0.089 *** (4.95)			
X_Efficacy*Mo_TaskSE		0.105 *** (6.31)		
X_InstGrat*Mo_SocialNorms			0.061 ** (3.41)	
X_Efficacy*Mo_SocialNorms				0.084 *** (4.94)
N	576	576	576	576

Table 3 displays unstandardized path coefficients from SEM testing the moderating effect on the relationship between predictors and overall AI dependency. Columns (1)–(2) present the results of moderator task-domain self-efficacy (*Mo_TaskSE*). Column (1) presents the model testing the moderation of the *X_InstGrat* effect. Column (2) presents the model testing the moderation of the *X_Efficacy* effect. Columns (3)–(4) present the results of moderator social norms (*Mo_SocialNorms*). Columns (3) presents the model testing the moderation of the *X_InstGrat* effect. Column (2) presents the model testing the moderation of the *X_Efficacy* effect. *AI_Dependency* is overall AI dependency; *X_Efficacy* is perceived AI efficacy; *X_InstGrat* is preference for Instant Gratification; *Mo_TaskSE* and *Mo_SocialNorms* are moderators, task-domain self-efficacy and social norms of AI use, respectively. Significance at the 1%, and 5%, levels is indicated by ***, **.

**Table 4:** Robustness Check.

	(1)		(2)	
	M_Erosion	AI_Dependency	M_Erosion	AI_Dependency
X_InstGrat	0.597 *** (13.08)	0.225 *** (6.00)		
X_Efficacy			0.621 *** (12.97)	0.349 *** (9.27)
Male	−0.101 (−1.59)	0.093 * (2.01)	−0.104 (−1.63)	0.074 (1.67)
M_Erosion		0.485 *** (16.13)		0.444 *** (15.39)
N	576	576	576	576

Table 4 presents the results of robustness check by including additional control variable, responder’s gender (*Male*). Column (1) presents the results of the *X_InstGrat* effect. Column (2) presents the results of the *X_Efficacy* effect. *AI_Dependency* is overall AI dependency; *X_Efficacy* is perceived AI efficacy; *X_InstGrat* is preference for Instant Gratification; *M_Erosion* is the mediator, erosion of self-efficacy; *Male* is a dummy variable equals one if the responder is a male, and zero otherwise. Significance at the 1% and 10% levels is indicated by *** and *, respectively.

## Data Availability

The data supporting the findings of this study are available from the corresponding author upon reasonable request due to ethical restrictions.

## Abbreviations

The following abbreviations are used in this manuscript:

AI	Artificial Intelligence
SEM	Structural Equation Modeling
SCT	Social Cognitive Theory
UTAUT	Unified Theory of Acceptance and Use of Technology
TAM	Technology Acceptance Model
